# Immunological dynamics associated with rapid virological response during the early phase of type I interferon therapy in patients with chronic hepatitis C

**DOI:** 10.1371/journal.pone.0179094

**Published:** 2017-06-14

**Authors:** Jae-Won Lee, Won Kim, Eun-Kyung Kwon, Yuri Kim, Hyun Mu Shin, Dong-Hyun Kim, Chan-Ki Min, Ji-Yeob Choi, Won-Woo Lee, Myung-Sik Choi, Byeong Gwan Kim, Nam-Hyuk Cho

**Affiliations:** 1 Department of Microbiology and Immunology, Seoul National University College of Medicine, Seoul, Republic of Korea; 2 Department of Biomedical Science, Seoul National University College of Medicine, Seoul, Republic of Korea; 3 Department of Internal Medicine, Seoul National University College of Medicine, Seoul Metropolitan Government Boramae Medical Center, Seoul, Republic of Korea; 4 Cancer Research Institute, Seoul National University College of Medicine, Seoul, Republic of Korea; 5 Institute of Endemic Disease, Seoul National University Medical Research Center and Bundang Hospital, Seoul, Republic of Korea; Korea Advanced Institute of Science and Technology, REPUBLIC OF KOREA

## Abstract

Type I interferons (IFNs) play an important role in antiviral immunity as well as immunopathogenesis of diverse chronic viral infections. However, the precise mechanisms regulating the multifaceted effects of type I IFNs on the immune system and pathological inflammation still remain unclear. In order to assess the immunological dynamics associated with rapid viral clearance in chronic hepatitis C patients during the acute phase of type I IFN therapy, we analyzed multiple parameters of virological and immunological responses in a cohort of 59 Korean hepatitis C patients who received pegylated IFN-α and ribavirin (IFN/RBV). Most of the Korean patients had favorable alleles in the IFN-λ loci for responsiveness to IFN/RBV (i.e., C/C in *rs12979860*, T/T in *rs8099917*, and TT/TT in *rs368234815*). Rapid virological response (RVR) was determined mainly by the hepatitis C virus genotype. Among the cytokines analyzed, higher plasma levels of IL-17A and FGF were observed in non-RVR patients infected with viral genotype 1 and IP-10 was consistently elevated in RVR group infected with genotype 2 during the early phase of antiviral therapy. In addition, these three cytokines were correlated each other, suggesting a functional linkage of the cytokines in antiviral responses during IFN/RBV therapy. A low baseline frequencies of regulatory T cells and γδ T cells, but high level of group 2 innate lymphoid cells, in peripheral bloods were also significantly associated with the RVR group, implicating a potential role of the cellular immunity during the early phase of IFN/RBV therapy. Therefore, the immunological programs established by chronic hepatitis C and rapid disruption of the delicate balance by exogenous type I IFN might be associated with the subsequent virological outcomes in chronic hepatitis C patients.

## Introduction

Type I interferons (IFNs) play a significant role in antiviral immunity as well as immunopathogenesis of chronic inflammatory diseases. The outcome of type I IFN responses during infectious disease is mediated by direct antiviral effects as well as immune modulatory functions on innate and adaptive immune systems [1]. Myriad studies have defined the regulatory effects of type I IFNs on innate immune cells such as dendritic cells, monocytes, and NK cells, which in turn regulate T cell-mediated immunity [2]. Type I IFN signaling enhances differentiation of monocytes into dendritic cells [3], promotes phenotypic maturation and migration of antigen-presenting cells (APCs) [4], and facilitates cross-presentation to CD8 T cells [5]. In addition, type I IFNs can enhance the cytotoxic activity of NK cells against virus-infected cells [6]. Moreover, type I IFNs contribute to viral clearance by acting directly on T cells, promoting their differentiation into IFN-γ-producing CD4 T cells [7] and enhancing the survival and clonal expansion of CD8 T cells [1]. However, an increasing amount of evidence shows that type I IFN responses can also result in detrimental outcomes during viral infection by inducing immunosuppressive effects and triggering inflammation and tissue damage [1,2]. Excessive type I IFN signaling in acute influenza infection can cause inflammatory monocytes-mediated epithelial cell death resulting in uncontrolled lung inflammation, which may be associated with severe morbidity [8]. Blocking IFN-mediated signaling also improves CD4 T cell-mediated virus control in lymphocytic choriomeningitis virus (LCMV) persistent infection [9]. Chronic LCMV infection induces sustained expression of type I IFNs in dendritic cells, which in turn upregulate suppressive immune modulators such as IL-10 and PD-L1 in APCs [9,10]. A recent study also reported that *in vivo* blockade of type I IFN signaling during chronic human immunodeficiency virus (HIV) infection diminished HIV-driven immune activation, decreased T cell exhaustion marker expression, restored HIV-specific CD8^+^ T cell function, and finally led to decreased viral replication [11]. Therefore, it is presumed that type I IFNs possess dual functions during viral infection: acute type I IFN responses contribute to antiviral and immune stimulation required for the clearance of viral infection, but sustained type I IFN signaling may induce immunosuppression that facilitates persistent viral infection. Nevertheless, the exact regulatory mechanism underlying the multifaceted effects of sustained type I IFN signaling on immunological mediators and pathological tissue damage during chronic viral infection remains elusive [12].

Due to their potent impact on antiviral immunity and immune modulatory functions to promote both pro-inflammatory and anti-inflammatory responses, type I IFNs have been widely used for the treatment of chronic viral infection such as hepatitis B virus (HBV), hepatitis C virus (HCV) [13], and HIV [14] infections. Although the advent of direct acting antivirals (DAAs) has spurred a trend toward type I IFN-free regimens in chronic hepatitis C (CHC), IFN therapy remains a significant therapeutic agent due to its antitumor efficacy against HCV-associated hepatocellular carcinoma (HCC) and an unexpected high recurrence rate of HCC after IFN-free therapy [12,15–17]. Given that the perturbation of the fine balance in immunological mediators by type I IFN treatment bears broad consequences ranging from hematopoiesis to control of adaptive immunity, understanding the basis underlying immune regulation is critical for improving the efficacy of current immunological therapies targeting type I IFN signaling [18]. In particular, the growing use of type I IFN therapy in HCV-infected patients since the early 1990s has provided valuable information on the virological and immunological impact of type I IFN on chronic viral infection, leading to the identification of several viral and host factors related to its treatment efficacy [13]. Although several host demographics such as age, sex, ethnicity, cirrhosis, steatosis, and body mass index (BMI) are known to be related to therapeutic efficacy, the major predictors of responsiveness to IFN/RBV therapy are viral genotypes containing genetic variations of several non-structural proteins such as NS5A [19]. However, the molecular basis by which these viral sequence variations affect responsiveness to antiviral therapy is largely unknown. Recently, genome-wide analysis has revealed that single nucleotide polymorphisms (SNPs) in the genetic locus encoding IFN-λ subtypes are associated with successful responses to IFN/RBV therapy [20]. Sustained elevation of IFN-stimulated genes during chronic HCV infection, potentially via prolonged IFN-λ expression, and dysregulation of HCV-specific adaptive immunity by T cell exhaustion and increased regulatory T cells (T_Reg_), are generally correlated with high viral loads and poor responsiveness to IFN/RBV therapy [20]. Moreover, increased Th17 cells as well as elevated IL-17 levels are also associated with severe liver damage [21]. Taken together, genetic variations of HCV and SNPs in host immunological factors such as IFN-λ are the major determinants of successful viral clearance upon exogenous perturbation of established inflammatory settings by type I IFN therapy. Although the causality between prolonged IFN expression and viral persistence, and how these predictors are associated with immunological dysfunction in chronic HCV infection are yet to be defined, the disruption of the delicate balance of immune factors by exogenous type I IFN during the early phase of treatment and subsequent viral clearance can provide valuable insight into IFN-related immunopathology and antiviral effects against HCV. To characterize the effect of exogenous type I IFN on innate and adaptive immune systems during the acute phase of therapy, and the immunological milieu that affects antiviral efficacy in CHC, we analyzed multiple parameters of virological and immunological responses in a prospective cohort of CHC patients who received IFN/RBV therapy.

## Materials and methods

### Study design and patients

We conducted a single-center prospective, cohort study evaluating the antiviral and anti-fibrotic effects of combination therapy of peginterferon α-2a plus ribavirin in Asian patients with CHC. This study included 59 consecutive CHC patients treated with IFN/RBV between 2013 and 2015 at the Seoul Metropolitan Government Seoul National University Boramae Medical Center. Rapid virological response (RVR) was defined as undetectable HCV RNA at 4 weeks after antiviral therapy. Baseline and clinical characteristics are presented in Table 1. Peripheral blood samples were collected before and one week after antiviral therapy and were used for immunological analysis. Written informed consent was obtained from all patients. This study was approved by the ethical committee of the Seoul Metropolitan Government Seoul National University Boramae Medical Center.

**Table 1:** Patients’ baseline characteristics.

	RVR (n = 36)	Non-RVR (n = 23)
Age, y	57.1 ± 10.7	58.0 ± 9.2
Male, n (%)	14 (23.7)	10 (43.5)
BMI	22.7 ± 3.1	25.1 ± 3.0
Fibrosis stage (Metavir), n (%)		
0 ~ 2	28 (77.8)	18 (78.3)
3 ~ 4	8 (22.2)	5 (21.7)
Steatosis grade, n (%)		
0 ~ 1	33 (91.7)	21 (91.3)
2	3 (8.3)	2 (8.7)
Biochemistry		
HDL (mg/dL)	52.2 ± 17.2	51.4 ± 21.8
LDL (mg/dL)	92.3 ± 24.2	167.3 ± 356.0
Glucose (mg/dL)	113.9 ± 38.1	117.1 ± 30.3
Cholesterol (mg/dL)	162.2 ± 29.7	163.8 ± 30.2
Triglycerides (mg/dL)	92.1 ± 28.8	97.3 ± 43.1
Total bilirubin (mg/dL)	0.8 ± 0.3	0.9 ± 0.4
AST (IU/L)	72.6 ± 45.0	48.5 ± 28.0
ALT (IU/L)	90.3 ± 73.3	50.4 ± 49.4
Insulin (μIU/dL)	14.6 ± 15.5	14.2 ± 10.4
HbA1c (%)	5.4 ± 0.9	5.8 ± 0.7
HOMA-IR	4.1 ± 4.9	4.3 ± 3.7
Platelet (counts/μL)	180.9 ± 60.9	183.5 ± 72.9
CRP (mg/dL)	0.1 ± 0.1	0.1 ± 0.0
SNPs in the IFN-λ locus, n (%)		
*rs12979860*		
C/C	32 (88.9%)	17 (73.9%)
C/T	4 (11.1%)	6 (26.1%)
*rs8099917*		
T/T	31 (86.1%)	17 (73.9%)
G/T	5 (13.9%)	6 (26.1%)
*rs368234815*		
TT/TT	36 (100%)	23 (100%)
HCV RNA titer Log_10_(IU/ml)	W0: 6.1 ± 6.3	W0: 6.4 ± 6.5
	W4: < 1	W4: 4.0 ± 4.4
HCV genotypes, n (%)		
1	6 (16.7%)	19 (82.6%)
2	30 (83.3%)	3 (13.0%)
6	0 (0%)	1 (4.4%)

Data are presented as mean ± SD.

BMI, body mass index; HDL, high-density lipoproteins; LDL, low-density lipoproteins; AST, aspartate aminotransferase; ALT, alanine aminotransferase; HbA1c, hemoglobin A1c; HOMA-IR, homeostatic model assessment-insulin resistance; CRP, C-reactive protein; SNP, single-nucleotide polymorphism.

### IFN-λ genotyping

*IFN-λ* genotypes (*rs8099917*, *rs12979860*, and *rs368234815*) [22] of the patients were determined by sequencing genomic DNAs extracted from peripheral blood mononuclear cells (PBMCs). The following primers were used for amplification and sequencing of the genetic loci; rs8099917 (forward: TCCCTCATCCCACTTCTGGA, reverse: CAAGTCAGGCAACCACATGC), rs12979860 (forward: GAAGGAGCAGTTGCGCTG, reverse: GAGGGACCGCTACGTAAGTC), and rs368234815 (forward: GGCAGGGCTCCCTTCTGTGATT, reverse: TGCCTTCCCTGGGATCCTAA).

### Virological, biochemical and clinical assessments

We obtained detailed information on age, sex, biochemistry, HCV genotype, and viral load from the electronic medical record. Baseline biochemical and hematological tests were done using automated techniques. Serological markers for viral hepatitis were detected using an automated chemiluminescent immunoassay system (ADVIA Centaur XP, Siemens, Erfurt, Germany). HCV RNA titers were quantitatively measured by reverse transcription polymerase chain reaction analysis using an Amplicor HCV amplification kit version 2.0 (Roche Diagnostic Systems, Basel, Switzerland). HCV was genotyped using an HCV Genotyping Chip kit version 2.0 (Biocore, Seoul, Korea) [23]. All study subjects underwent liver biopsy within one month before the initiation of antiviral therapy. Histological assessment of liver biopsy specimens was performed by a single expert pathologist blinded to the clinical data. The META-analysis VIRus hepatitis histologic scoring system (METAVIR) was used to evaluate the fibrosis stage. The grade of hepatic steatosis was assessed using the Kleiner scoring system [24].

### Preparation of plasma and PBMCs

Blood samples were collected from each patient twice, before and one week after IFN/RBV treatment, in sodium heparin tubes (BD Biosciences, San Jose, CA). Plasma and PBMCs were isolated using Ficoll-Paque plus (GE Healthcare, Wauwatosa, WI) density gradient centrifugation. Isolated plasma and PBMCs were stored at -80℃ and –196℃, respectively, until used.

### Cytokine analysis

Concentrations of cytokines (IL-2, IL-4, IL-6, IL-10, IL-12(p70), IL-17, IFN-γ, and TNF-α) and growth factors (FGF and VEGF) were measured by using Bio-Plex Pro^TM^ cytokine, chemokine, and growth factor assay (Bio-Rad, Hercules, CA). Plasma concentrations of thymic stromal lymphopoietin (TSLP), interferon γ-induced protein 10 (IP-10) (Biolegend, San Diego, CA), platelet factor 4 (PF4) (Sigma-Aldrich, St. Louis, MO), and serotonin (Abcam, Cambridge, UK) were measured by ELISA in accordance with the manufacturer’s instructions.

### Flow cytometric analysis

Relative frequencies of diverse immune cells in patients’ PBMCs were analyzed after staining with several antibodies. Allophycocyanin-cyanine7 (APC/Cy7)-conjugated anti-CD3 (BD Biosciences), phycoerythrincy-cyanine5 (PE/Cy5)-conjugated anti-CD4 (BD Biosciences), PE-conjugated anti-γδ T cell receptor (Biolegend), APC-conjugated anti-CD56 (BD Biosciences), and fluorescein isothiocyanate (FITC)-conjugated anti-IL-17A (eBioscience, Hatfield, UK) were used for identifying CD4 T cells, γδ T cells, and NK cells. FITC-conjugated anti-human lineage cocktail2 (Lin2) (BD Biosciences), PE/Cy7-conjugated anti-CD45 (eBioscience), Alexa700-conjugated anti-IL-17A (Biolegend), brilliant violet 421-conjugated anti-IL-7R (Biolegend), and PE-conjugated anti-IL-13 (Biolegend) were used for analyzing group 2 innate lymphoid cell **(**ILC2) and group 3 innate lymphoid cell **(**ILC3). Alexa700-conjugated anti-CD8 (BD Biosciences), PE/Cy7-conjugated anti-CD19 (Biolegend), Pacificblue-conjugated anti-CD4 (Biolegend), FITC-conjugated anti-CD25 (Biolegend), and APC-conjugated anti-FOXP3 (eBioscience) were used for detecting T_Reg_. To detect IL-17A or IL-13-positive cells, PBMCs were incubated with 100 ng/mL phorbol myristate acetate (PMA), 1 μg/mL ionomycin, and Golgistop (BD Biosciences) at 37℃ for 5 h. Intracellular staining was performed using the Cytofix/Cytoperm™ Kit (BD Biosciences) or a fixation and permeabilization buffer set (eBioscience) according to the manufacturer’s instructions. The staining process was performed using flow cytometry buffer (0.5% BSA in PBS) with Fc Block (2.4G2, BD Biosciences). The stained cells were analyzed on a BD LSRFortessa X-20 or LSR II (BD Biosciences) and the data were analyzed with FlowJo software (TreeStar Inc., Ashland, OR). All the cells were gated first for lymphocytes (FSC-A and SSC-A) and singlets (FSC-A and FSC-H). CD4^+^ T cells were gated on CD3^+^ CD4^+^ population, and γδ T cells were gated on γδ TCR^+^ cells among CD3^+^ cells. NK cells were gated on CD56^+^ population. ILC2 and ILC3 were detected by Lin^-^/IL-7R^+^/CD45^+^/IL-13^+^ and Lin^-^/IL-7R^+^/CD45^+^/IL-17A^+^, respectively. T_Reg_ cells were gated on CD8^-^/CD4^+^/CD25^+^/CD19^-^/FoxP3^+^ cells. Representative gating strategies for each cellular population are presented in S1 Fig.

### Statistical analysis

Student’s *t*-test or Mann-Whitney U test was used to compare statistical differences between the two groups. All the raw data sets used in this study are presented in S1 Table. One-way analysis of variance (ANOVA) followed by Newman-Keuls *t*-test was performed for comparisons of the values among different groups. Associations between variables were evaluated using Spearman rank-order correlation. Multivariate logistic regression analysis was applied to detect independent predictors of RVR. Data analysis was performed using SPSS software version 16.0 (SPSS Inc., Chicago, IL) and GraphPad Prism 5.0 software (GraphPad Software Inc., San Diego, CA). *P* < 0.05 was considered statistically significant.

## Results

### Comparison of baseline characteristics between the RVR and non-RVR groups

Among the 59 patients, 36 (61%) patients attained RVR after IFN/RBV therapy, whereas 23 (39%) patients did not achieve RVR (Table 1). The RVR group had significantly lower BMI (*p* = 0.006) and higher aspartate aminotransferase (AST) and alanine aminotransferase (ALT) levels (*p* = 0.026 in both parameters) prior to IFN/RBV therapy compared to the non-RVR group. There were no significant differences in other clinical and biochemical parameters between the two groups (Table 1). We also examined patients’ IFN-λ SNPs since they are significantly associated with spontaneous viral clearance and virological response to IFN/RBV treatment in some ethnic populations [25]. However, the relative proportions of major/minor alleles in three SNPs (*rs12979860*, *rs8099917*, *and rs368234815*) in the IFN-λ loci were not significantly different between the RVR and non-RVR groups. Thirty (83.3%) patients in the RVR group were infected with HCV genotype 2, while 19 (82.6%) patients in the non-RVR had genotype 1. Baseline viral load of HCV was slightly higher in the non-RVR group than in the RVR group (*p* = 0.0391).

### Plasma IL-17A, IP-10, and FGF levels are associated with RVR upon IFN/RBV treatment

It has been reported that a group of cytokines involved in innate and adaptive immune responses are elevated in CHC patients [20,26]. Therefore, we investigated whether IFN/RBV treatment affects cytokine responses during the first week of therapy. Although we could not detect significant changes in any of the cytokines during the first week of treatment (Table 2), there were significant differences in the levels of several cytokines and growth factors between the RVR and non-RVR groups. IL-17A levels remained relatively higher in patients with non-RVR, whereas IP-10 levels were higher in the RVR group before and one week after antiviral therapy. In addition, FGF, a mediator of fibrogenesis induction, was significantly elevated in patients with non-RVR than in those with RVR during the early phase of treatment.

**Table 2:** Comparison of cytokine responses between the RVR and non-RVR groups.

	RVR		non-RVR	
	Week 0	Week 1	Week 0	Week 1
IL-2, n	30.4 ± 73.7,	36.1 ± 69.8,	25.9 ± 48.0,	31.6 ± 50.9,
	*n = 17*	*n = 17*	*n = 16*	*n = 16*
IL-4, n	1.5 ± 2.1,	2.0 ± 3.2,	1.5 ± 1.8,	1.8 ± 1.8,
	*n = 17*	*n = 17*	*n = 16*	*n = 16*
IL-6, n	26.8 ± 52.2,	30.9 ± 49.8,	18.5 ± 15.5,	60.6 ± 148.8,
	*n = 17*	*n = 17*	*n = 16*	*n = 16*
IL-10, n	18.2 ± 18.5,	25.4 ± 36.0,	20.9 ± 14.0,	24.4 ± 17.0,
	*n = 17*	*n = 17*	*n = 16*	*n = 16*
IL-12(p70), n	122.2 ± 163.8,	130.5 ± 178.7,	113.7 ± 110.5,	122.9 ± 200.0,
	*n = 17*	*n = 17*	*n = 16*	*n = 16*
IL-17, n	90.8 ± 103.1,	97.7 ± 176.9,	405.4 ± 235.6,	403.3 ± 244.1,
	*n = 23*	*n = 23*	*n = 18*	*n = 18*
IFN-γ, n	44.0 ± 92.5,	62.9 ± 117.3,	75.6 ± 131.4,	76.4 ± 131.4,
	*n = 17*	*n = 17*	*n = 16*	*n = 16*
TNF-α, n	22.8 ± 25.2,	30.6 ± 36.0,	33.4 ± 87.1,	45.2 ± 98.5,
	*n = 17*	*n = 17*	*n = 16*	*n = 16*
TSLP, n	16.4 ± 45.0,	20.9 ± 66.2,	7.4 ± 6.6,	8.0 ± 6.4,
	*n = 21*	*n = 21*	*n = 16*	*n = 16*
IP-10, n	540.9 ± 278.4,	569.6 ± 299.7,	301.3 ± 194.6,	311.3 ± 142.4,
	*n = 31*	*n = 31*	*n = 20*	*n = 20*
PF4, n	204.6 ± 153.5,	248.8 ± 188.8,	195.1 ± 143.7,	185.1 ± 119.2,
	*n = 21*	*n = 21*	*n = 16*	*n = 16*
Serotonin, n	12.5 ± 8.3,	11.5 ± 3.4,	11.7 ± 4.4,	12.2 ± 8.8,
	*n = 21*	*n = 21*	*n = 16*	*n = 16*
FGF, n	57.3 ± 62.2,	60.1 ± 92.6,	125.2 ± 81.6,	130.9 ± 84.3,
	*n = 23*	*n = 23*	*n = 18*	*n = 18*
VEGF, n	368.9 ± 242.4,	420.3 ± 319.3,	320.0 ± 162.9,	327.8 ± 194.7,
	*n = 23*	*n = 23*	*n = 18*	*n = 18*

Data (pg/ml) are presented as mean ± SD.

IL, interleukin; IFN, interferon; TNF, tumor necrosis factor; TSLP, thymic stromal lymphopoietin; IP-10, interferon γ -induced protein 10; PF4, platelet factor 4; FGF, fibroblast growth factor; VEGF, vascular endothelial growth factor.

**Fig 1:**
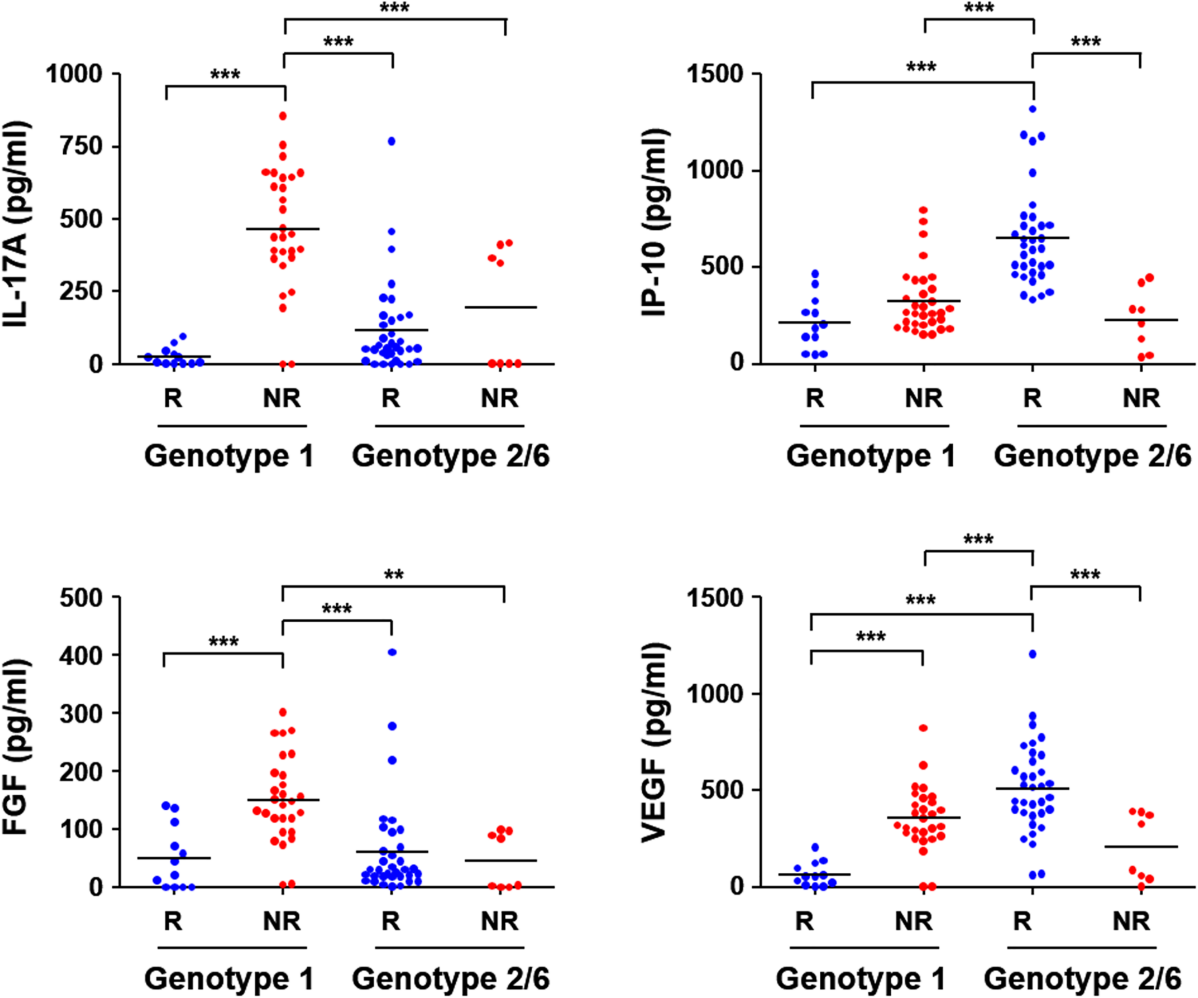
Differential expression of cytokines in the plasma of chronic hepatitis C patients infected with different viral genotypes. The levels of IL-17A, IP-10, FGF, and VEGF in patients’ plasma were examined. The plasma concentrations of four cytokines that were differentially expressed in RVR (R) and non-RVR (NR) patients infected with genotype 1 or genotype 2/6 are presented. The data points (RVR with genotype 1: *n* = 12, non-RVR with genotype 1: *n* = 32, RVR with genotype 2: *n* = 34, non-RVR with genotype 2/6: *n* = 8) were derived from patients’ samples before and one week after type I IFN therapy. Horizontal bars represent the mean values. **, *p* < 0.01; ***, *p* < 0.001.

In order to assess the effect of viral genotypes on the cytokine responses, we also compared the levels of cytokines in the RVR and non-RVR groups during the early phase of antiviral treatment (Fig 1). The levels of IL-17A and FGF measured before and one week after the therapy showed similar patterns when they were differentiated based on the viral genotypes infected. Both cytokines were significantly lower in RVR patients than those of non-RVR group when they were infected with genotype 1, whereas there was no significant difference in the level of cytokines between RVR and non-RVR patients infected with genotype 2 or 6. Interestingly, the plasma levels of two cytokines, IP-10 and VEGF, were differentially observed in RVR and non-RVR patients when they were infected with different viral genotypes. They were relatively higher in non-RVR patients than in RVR patients when infected with genotype 1, whereas the trends were reversed if they were infected with genotype 2 or 6 (Fig 1). The levels of IP-10 and VEGF were significantly higher in RVR group infected with genotype 2/6 than those of patients infected with genotype 1 and non-RVR patients infected with genotype 2/6. These results suggest that the rapid viral responses during the acute phase of type I IFN therapy might be associated with the viral genotypes infected as well as the levels of specific cytokines.

**Fig 2:**
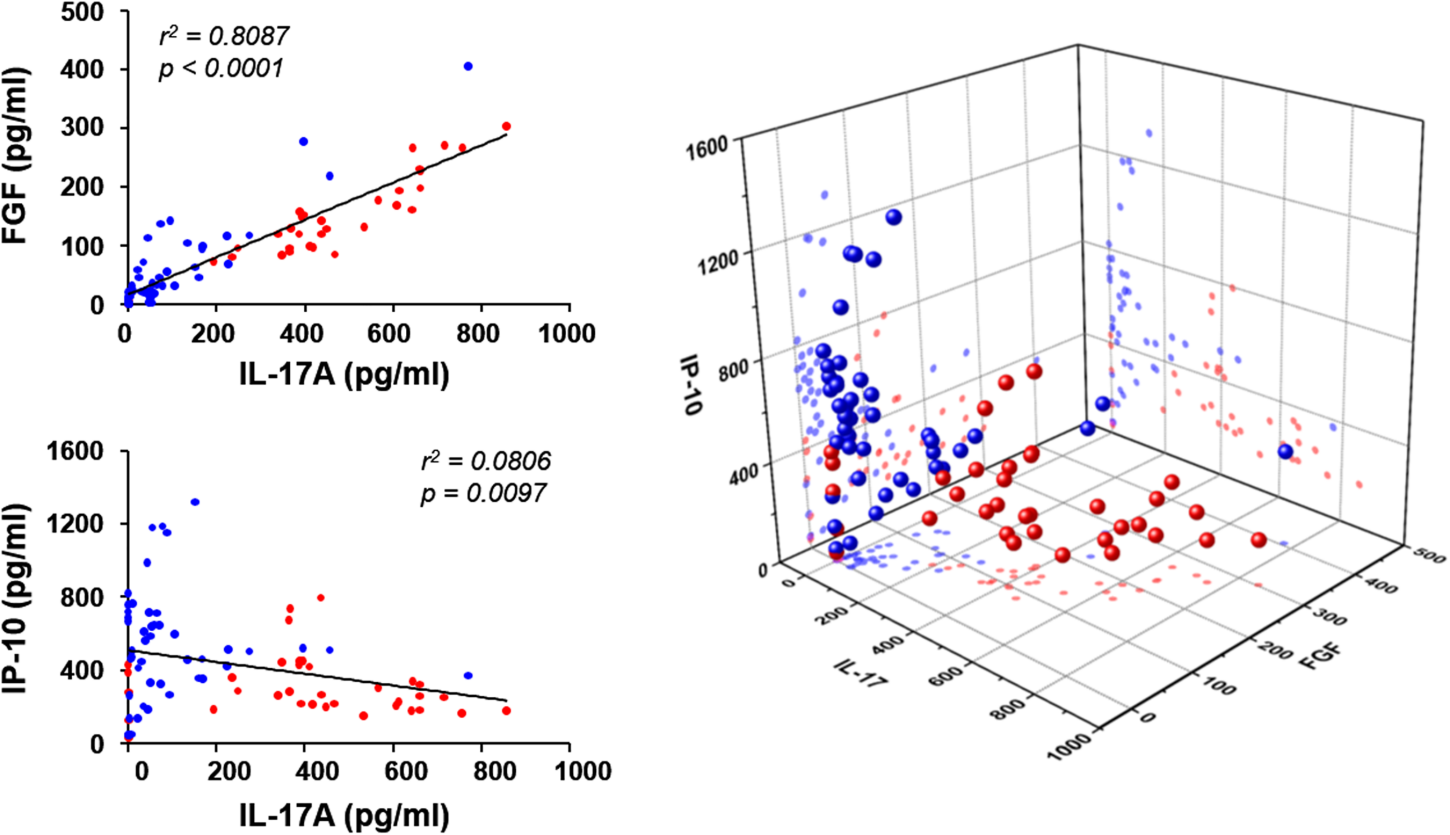
Correlation of IL-17A, IP-10, and FGF levels in patients’ plasma. The plasma levels of IL-17A were correlated with FGF or IP-10 levels in either a positive or negative way (left two panels). The correlations among the three cytokines are also presented in 3D plot (right panel). The data points (*n* = 66) derived from patients with RVR (blue dots) and non-RVR (red dots) before and one week after type I IFN therapy are indicated.

We also assessed potential correlations among the cytokines differentially expressed (Fig 2). The level of IL-17A measured before and one week after type I IFN therapy was positively correlated with the FGF level (*p* < 0.001) and negatively correlated with the IP-10 level (*p* < 0.001). In addition, the plasma IP-10 level was also negatively correlated with the FGF level (*p* < 0.001). These results suggest that these cytokines might be closely associated with the immunological environment that affects the efficacy of viral clearance during IFN/RBV treatment in CHC patients.

### The frequencies of IL-17A^+^ CD4 T cells are associated with the plasma IL-17A levels

Next, we assessed leukocyte counts before and one week after treatment (Table 3). Total counts of leukocyte were generally reduced in both the RVR and non-RVR groups upon IFN/RBV treatment, primarily due to the rapid decline in neutrophil counts regardless of the viral genotypes infected. General reductions in the frequencies of eosinophil and basophil were also observed in all the groups, except non-RVR patients infected with genotype 2 or 6, during the first week after IFN/RBV treatment, whereas lymphocyte and monocyte counts were not significantly changed during the early phase of treatment (Table 3).

**Table 3:** Comparison of leukocyte counts between the RVR and non-RVR groups.

	RVR				Non-RVR			
	Genotype 1		Genotype 2/6		Genotype 1		Genotype 2/6	
	Week0	Week1	Week0	Week1	Week0	Week1	Week0	Week1
White blood cells,counts/μL	5,470 ± 1,286	4,387 ± 1,745	5,347 ± 1,353	5,214 ± 959	5,318 ± 2,030	4,076 ± 1,494	5,048 ± 1,083	3,927 ± 1,716
Lymphocytes, counts/μL (%)	2,206 ± 971	2,223 ± 1,296	2,250 ± 719	3,140 ± 759	2,439 ± 1,044	1,491 ± 846	1,976 ± 303	1,980 ± 595
	(39.1 ± 8.0)	(48.8 ± 8.9)	(41.7 ± 5.0)	(59.7 ± 5.9)	(47.6 ± 4.7)	(34.4 ± 8.2)	(40.1 ± 7.3)	(52.4 ± 9.8)
Monocytes, counts/μL (%)	372 ± 132	363 ± 152	378 ± 141	363 ± 61	329 ± 144	303 ± 183	314 ± 42	276 ± 165
	(6.7 ± 1.2)	(8.3 ± 1.4)	(7.0 ± 1.9)	(7.3 ± 2.4)	(6.4 ± 1.2)	(6.9 ± 1.2)	(6.4 ± 1.5)	(6.9 ± 2.8)
Neutrophils, counts/μL (%)	2,708 ± 317	1,712 ± 491	2,534 ± 633	1,616 ± 389	2,439 ± 1,044	1,491 ± 846	2,562 ± 754	1,410 ± 638
	(51.0 ± 8.4)	(41.1 ± 9.9)	(48.0 ± 7.4)	(31.2 ± 6.2)	(47.6 ± 4.7)	(34.4 ± 8.2)	(50.1 ± 7.2)	(35.9 ± 4.4)
Eosinophils, counts/μL (%)	148 ± 121	79 ± 69	164 ± 124	88 ± 29	144 ± 153	99 ± 93	185 ± 243	250 ± 382
	(2.6 ± 2.1)	(1.8 ± 1.5)	(2.9 ± 1.7)	(1.7 ± 0.4)	(2.7 ± 2.6)	(2.1 ± 1.3)	(3.2 ± 3.9)	(4.6 ± 6.2)
Basophils, counts/μL (%)	36 ± 20	8 ± 12	20 ± 15	7 ± 6	21 ± 34	9 ± 6	11 ± 8	10 ± 17
	(0.7 ± 0.4)	(0.2 ± 0.2)	(0.4 ± 0.2)	(0.1 ± 0.1)	(0.4 ± 0.6)	(0.2 ± 0.2)	(0.2 ± 0.1)	(0.2 ± 0.3)

Data are presented as mean ± SD.

**Fig 3:**
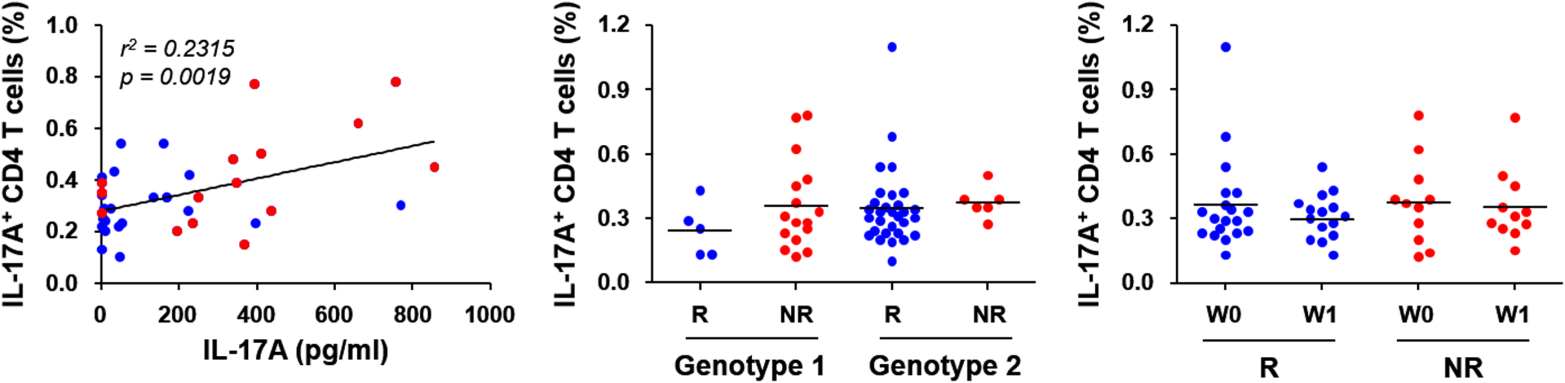
Correlation of plasma IL-17A levels with Th17 cells in peripheral blood. The ratio of IL-17A^+^ CD4 T cells (Th17) per CD4 T cells was positively correlated with plasma IL-17A levels. The data points (*n* = 39) derived from patients with RVR (blue dots) and non-RVR (red dots) before and one week after type I IFN therapy are indicated (left panel). The proportions (%) of IL-17A^+^ CD4 T cells (Th17) among peripheral CD4 T cells in patients with RVR (R) and non-RVR (NR) infected with viral genotype 1 or genotype 2 (middle panel) before (W0) and one week (W1) after type I IFN therapy (right panel) are also analyzed. Horizontal bars represent the mean values.

**Fig 4:**
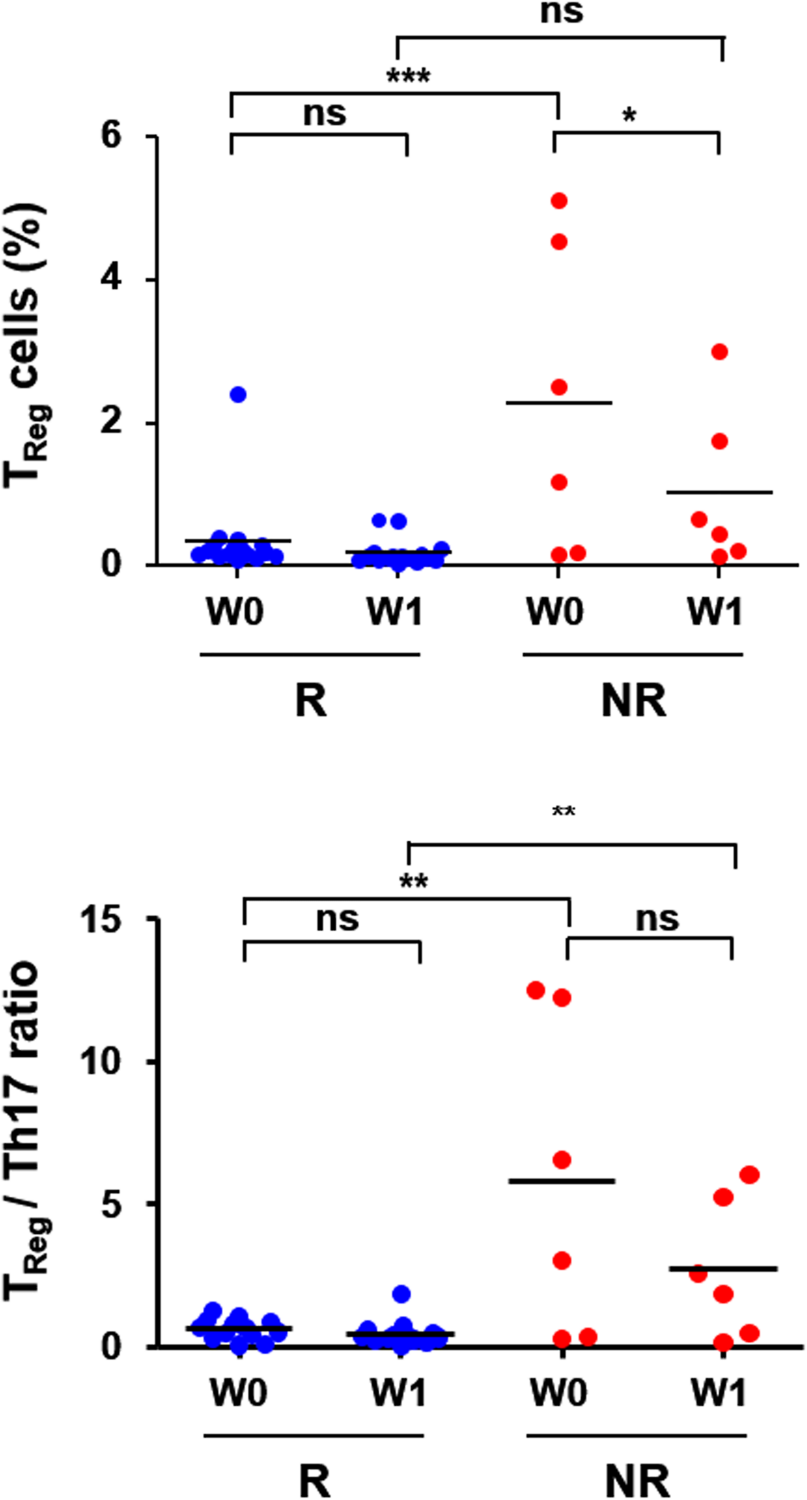
Relative frequencies of Th17 and T_Reg_ cells in peripheral blood of chronic hepatitis C patients. The proportions (%) of CD4^+^CD25^+^FoxP3^+^ T cells (T_Reg_) among peripheral CD4 T cells in patients with RVR (R) and non-RVR (NR) before (W0) and one week (W1) after type I IFN therapy are presented (upper panel). T_Reg_: Th17 ratio was also presented in the lower panel. Horizontal bars represent the mean values. *, *p* < 0.05; **, *p* < 0.01; ***, *p* < 0.001. ns, not significant.

Since the plasma levels of IL-17A and Th17 cells have been known to be associated with the severity of liver inflammation and/or virological response upon IFN/RBV therapy [21,27,28], we analyzed the potential correlation of the plasma level of IL-17A with the frequency of Th17 cells secreting IL-17A. Based on flow cytometric analysis, the proportion of IL-17A^+^ CD4 T cells in PBMCs was correlated with the plasma concentration of IL-17A measured before and one week after type I IFN therapy (Fig 3, left panel), suggesting that Th17 cells might be the primary source of plasma IL-17A. However, there were no notable differences in the proportion of IL-17A^+^ CD4 T cells as well as CD4 T cells in PBMCs between the RVR and non-RVR groups infected with different viral genotypes before and one week after antiviral therapy (Table 4 and Fig 3, middle and right panel). Instead, we could observe a significantly higher level of CD4^+^CD25^+^FoxP3^+^ T_Reg_ cells in the non-RVR group than in the RVR group before IFN/RBV treatment (Table 4 and Fig 4). Interestingly, the proportion of T_Reg_ cells was significantly reduced in patients with non-RVR from baseline to one week treatment.

**Table 4:** Comparison of PBMC populations between the RVR and non-RVR groups.

	RVR		non-RVR	
	Week 0	Week 1	Week 0	Week 1
CD4^+^ T cells (%), n	22.6 ± 6.3,	24.5 ± 8.1,	22.4 ± 6.5,	21.1 ± 6.5,
	*n = 20*	*n = 20*	*n = 11*	*n = 11*
IL-17^+^ CD4^+^ T cells (%), n	0.4 ± 0.2,	0.3 ± 0.1,	0.4 ± 0.2,	0.4 ± 0.2,
	*n = 19*	*n = 16*	*n = 11*	*n = 11*
CD8^+^ T cells (%), n	11.6 ± 4.3,	11.6 ± 3.8,	11.7 ± 5.5,	12.8 ± 5.5
	*n* = 20	*n* = 20	*n* = 11	*n* = 11
IL-17^+^ CD8^+^ T cells (%), n	0.16 ± 0.13	0.17 ± 0.16	0.20 ± 0.23	0.19 ± 0.21
	*n* = 20	*n* = 20	*n = 11*	*n = 11*
T_Reg_ (%), n	0.3 ± 0.6,	0.2 ± 0.2,	2.3 ± 2.2,	1.0 ± 1.1,
	*n = 16*	*n = 16*	*n = 6*	*n = 6*
γδ T cells (%), n	2.1 ± 1.3,	2.8 ± 1.3,	3.8 ± 2.3,	4.6 ± 2.2,
	*n = 20*	*n = 20*	*n = 11*	*n = 11*
IL-17^+^ γδ T cells (%), n	0.05 ± 0.03,	0.04 ± 0.02,	0.05 ± 0.05,	0.04 ± 0.03,
	*n = 19*	*n = 16*	*n = 11*	*n = 11*
NK cells (%), n	7.6 ± 6.3,	8.3 ± 5.1,	8.0 ± 5.5,	9.4 ± 5.0,
	*n = 20*	*n = 20*	*n = 11*	*n = 11*
IL-17^+^ NK cells (%), n	0.1 ± 0.1,	0.2 ± 0.2,	0.2 ± 0.2,	0.1 ± 0.1,
	*n = 19*	*n = 16*	*n = 11*	*n = 11*
ILC2 (%), n	0.11 ± 0.03,	0.12 ± 0.04,	0.08 ± 0.04,	0.06 ± 0.03,
	*n = 23*	*n = 23*	*n = 11*	*n = 11*
ILC3 (%), n	0.05 ± 0.02,	0.05 ± 0.02,	0.03 ± 0.03,	0.02 ± 0.02,
	*n = 23*	*n = 23*	*n = 11*	*n = 11*

Data are presented as mean ± SD.

NK, natural killer; ILC, innate lymphoid cell.

### Differential responses of γδ T cells and innate lymphoid cells in HCV-infected patients

**Fig 5:**
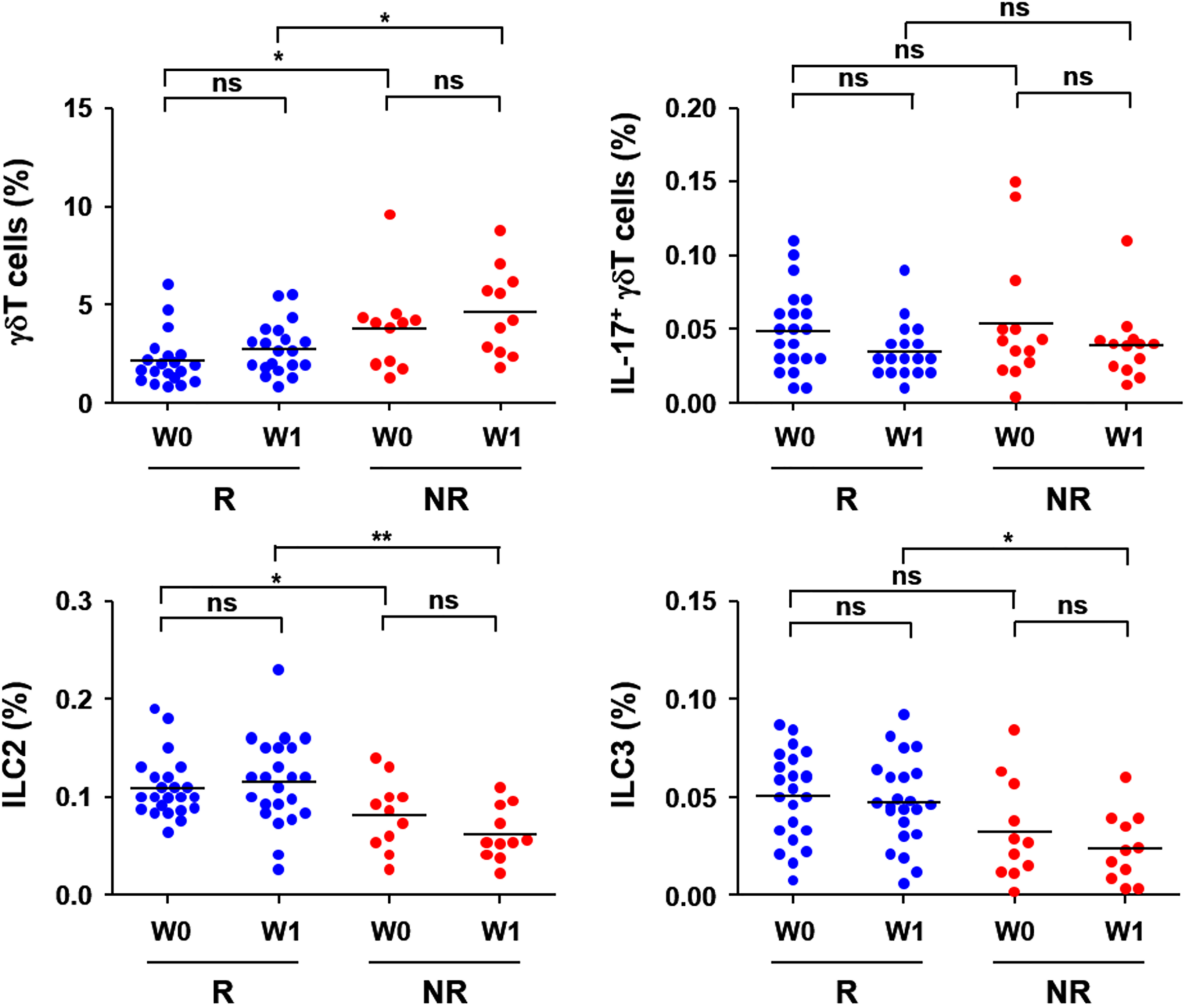
Relative frequencies of innate immune cells in peripheral blood of chronic hepatitis C patients. The proportions (%) of γδ T cells, IL-17A^+^ γδT cells, and group 2 and group 3 innate lymphoid cells (ILC2 and ILC3) in PBMCs of patients with RVR (R) and non-RVR (NR) before (W0) and one week (W1) after type I IFN therapy are presented. Horizontal bars represent the mean values. *, *p* < 0.05; **, *p* < 0.01; ***, *p* < 0.001.

In addition to Th17 cells, diverse ILCs including NK cells, γδ T cells, and ILC3 also produce IL-17 [29,30]. To assess potential differences in innate lymphoid responses, we examined the frequencies of NK cells, γδ T cells, ILC2, and ILC3 in PBMCs collected from CHC patients (Table 4 and Fig 5). There were no significant differences in the frequency of NK cells before and one week after IFN/RBV therapy between the RVR and non-RVR groups. However, the frequency of γδ T cells was significantly higher in the non-RVR group than in the RVR group, and was slightly elevated upon antiviral therapy in both groups although it did not reach statistical significance. In contrast, the proportion of IL-17A^+^ γδ T cells was not statistically different between the two groups and was marginally reduced without statistical significance upon IFN/RBV therapy. The frequencies of ILC2 and ILC3 which secrete IL-13 and IL-17, respectively, were generally lower in the non-RVR group than in the RVR group, but no significant changes in their frequencies were observed during the early phase of antiviral therapy.

## Discussion

Although the recent advance in anti-HCV therapies using DAAs opens a new avenue to treating CHC, the application of type I IFN therapy during the last few decades has provided valuable insight into the immunomodulatory functions of type I IFN and is a good model system for studying the complex interplay between innate and adaptive immunity in humans. Since the degree of virological response upon type I IFN treatment is variable and determined by a variety of host and viral factors, and the early kinetics of virological response are the major predictors of sustained virological response (SVR) [13,31], the immunological programs established by chronic HCV infection might play a pivotal role in the efficient viral clearance upon exogenous type I IFN. To elucidate the regulatory mechanisms of the innate cytokines in altering antiviral immunity, we investigated the early dynamics of diverse immunological parameters from baseline to one week treatment with type I IFN.

The study subjects enrolled in this study were allocated into the RVR and non-RVR groups based on the early viral kinetics. These two groups showed significant differences in several clinical characteristics before antiviral therapy. The mean BMI of the non-RVR group was significantly higher than that of the RVR group, whereas other metabolic parameters such as the grade of steatosis and the plasma levels of lipids were similar in both groups. Indeed, high BMI is an independent risk factor for poor responsiveness to antiviral treatment [32], although the causality between BMI and RVR to IFN/RBV therapy is still unclear. We also observed the elevated AST and ALT levels, representing severe liver inflammation, in patients with RVR and higher pretreatment levels were independently associated with RVR. A significant correlation of baseline ALT levels with RVR was already reported [33], but this has not been consistently observed [28], suggesting the degree of liver inflammation may not be a major determinant of RVR.

SNPs in the IFN-λ loci are associated with spontaneous viral clearance and SVR following IFN/RBV therapy, and the ethnicity-dependent distribution of HCV genotypes partially explains the differences in virological responsiveness [22]. As shown in the previous reports [34,35], most Korean patients have favorable SNPs in the IFN-λ loci (i.e., CC genotype in *rs12979860* and TT in *rs8099917*). In addition, all Korean patients examined in this study possess a recently identified *rs368234815*‑TT/TT allele, which does not generate IFN-λ4 because of a premature stop codon and is associated with better virological response to IFN/RBV therapy [36]. Due to the prevalence of favorable genotypes for IFN-based therapy, the SVR rates among Korean patients are relatively higher than those among other ethnic populations, regardless of the viral genotype [37]. Nevertheless, relative proportions of the three SNPs analyzed in this small-scale study were similar between the RVR and non-RVR groups, suggesting that IFN-λ SNPs might not be useful predictors of RVR upon IFN/RBV therapy in Korean CHC patients [35].

Differing from IFN-λ SNPs, viral genotypes are significantly correlated with RVR (Table 1). Patients infected with viral genotype 2 showed rapid viral clearance, whereas those infected with genotype 1 mostly had less efficient virological response to IFN/RBV therapy. Additionally, when we analyzed the diverse cytokine responses, only the levels of IL-17A, IP-10, and FGF among a total of 11 cytokines were significantly associated with RVR in a viral genotype-dependent manner (Table 2 and Fig 1). Baseline levels of several cellular factors such as TSLP [38] and serotonin [39], were also poorly correlated with early virological outcomes upon treatment. Lower IL-17A, FGF, and VEGF levels were differentially measured in the plasma of RVR patients than in that of the non-RVR group when infected with genotype 1. However, higher levels of IP-10 and VEGF levels were consistently observed in RVR patients than in non-RVR group infected with genotype 2 or 6 (Fig 1). Notably, the plasma concentrations of these cytokines were also correlated with each other, indicating a functional linkage of the cytokines in the immunological niche involved in the antiviral responses before and during the acute phase of IFN/RBV therapy (Fig 2). Although the significant elevation of these cytokines in CHC patients than in healthy controls has been previously reported [21,26,40], such a reciprocal correlation has never been reported before. Instead, there have been contradictory results to our current study. IL-17A has been implicated in the host defense against microbial pathogens but is also associated with inflammatory disorders when produced in excess [30,41], and the cytokine levels were correlated with serum ALT levels or viral copy numbers in CHC patients [21,42]. However, IL-17A was not significantly elevated in CHC patients when compared to the healthy control group in other studies [28,43]. We observed significant elevation of IL-17A levels only in non-RVR patients infected with HCV genotype 1 (Fig 1). IP-10 has been also suggested to be a predictive marker of RVR and SVR in patients infected with viral genotype 1 [40,44], but it was not significantly correlated with virological response in Korean patients with CHC [45]. Even though the levels of IP-10 in RVR patients were slightly lower without statistical significance than in non-RVR group when infected with genotype 1, higher baseline IP-10 levels were positively associated with RVR when patients were infected with genotype 2 or 6 in the current study, suggesting a complex relationship of cytokines and acute virological response upon IFN/RBV therapy depending on the viral genotypes. Such differences can also be attributable to the number of enrolled subjects and the distribution of liver injury severity. Nevertheless, it is noteworthy that the levels of IL-17A were positively correlated with FGF levels, but negatively associated with IP-10 levels, suggesting a unique immunological milieu dictated by the viral genotype. Considering that IL-17A, FGF, and IP-10 are associated with liver inflammation stem from Th17 responses [43], tissue repair [46], and recruitment of inflammatory cells during Th1 responses [47], respectively, immunological settings with lower Th17 responses accompanying less fibrogenic environment, as well as enhanced Th1-biased immunity, may favor the RVR during the acute phase of IFN IFN/RBV therapy. In addition, the baseline levels of the four cytokines, IL-17A, FGF, IP-10, and VEGF, can be predictive of the response to the clinical intervention in CHC patients depending on the infected HCV genotypes (Fig 1). The functional relationship between the cytokine levels and types of helper T cell responses needs to be further characterized.

Because we observed a unique correlation between baseline IL-17A levels and rapid viral clearance upon IFN/RBV therapy, potential sources of IL-17A were examined using patients’ PBMCs. The plasma levels of IL-17A were significantly correlated with cellular frequencies of the IL-17A^+^ CD4 T cells in PBMCs (Fig 3), indicating that Th17 cells might be the primary source of IL-17A as reported in the previous study [21]. However, relative frequencies of IL-17A^+^ CD4 T cells of the RVR group were similar to those of the non-RVR group and not significantly affected by HCV genotypes (Fig 3). This might be due to the differences in the gene expression levels of IL-17A in Th17 cells, which merits further scrutiny. Conversely, the proportion of T_Reg_ was significantly higher in the non-RVR group than in the RVR group and was reduced one week following antiviral therapy, which was in accord with the previous study [28]. Therefore, the balance between T_Reg_ and Th17 (i.e., the ratio of T_Reg_ to Th17 cells, Fig 4) during chronic HCV infection might play a crucial role in acute viral clearance upon IFN/RBV therapy. The plasticity and the reciprocal relationship between T_Reg_ and Th17 development have also been implicated in disease progression and outcomes of chronic inflammatory disorders [48].

Inconsistency between higher plasma levels of IL-17A in non-RVR patients and similar frequencies of Th17 cells in both groups prompted us to investigate relative proportions of other immune cells that produce IL-17 during inflammatory conditions such as CD8 T cells, NK cells, γδ T cells, and innate lymphoid cells (ILCs), [30]. Despite extensive cellular analysis of patients’ PBMCs, there were no significant correlations of IL-17A levels with relative frequencies of the immune cells (Table 4 and Fig 5). In addition, the proportion of these cells was not significantly changed during the first week of antiviral therapy. Rather, there were slightly higher γδ T cells and lower ILCs in PBMCs of the non-RVR group than in those of the RVR group. Although polymorphonuclear cells including neutrophils rapidly decreased during the first week of IFN/RBV therapy, relative frequencies of innate immune cells were not significantly changed in both groups (Fig 5 and Table 3). Taken together, peripheral frequencies of innate and adaptive immune cells, except γδ T cells, ILCs, and T_Reg_ cells, were not significantly different between the RVR and non-RVR groups before antiviral treatment and there were marginal changes in the populations of innate immune cells during the acute phase of IFN/RBV therapy. Although γδ T cells [49] and ILCs [50] have been implicated in liver inflammation during the evolution of CHC, their specific role in antiviral immunity and inflammatory damage in CHC remains controversial or has been poorly defined. Therefore, further studies on their contribution to the pathogenesis of CHC and therapeutic outcomes may provide important information on the immunological program established by chronic HCV infection and for the development of better strategies against CHC.

## Supporting information

S1 Fig Gating strategies for the analysis of diverse immune cell types in peripheral blood mononuclear cells of chronic hepatitis C patients. (PDF)

S1 Table Raw data sets used in this study. (XLS)

## References

[1] McNab F, Mayer-Barber K, Sher A, Wack A, O'Garra A (2015) Type I interferons in infectious disease. Nat Rev Immunol 15: 87–103. doi: 10.1038/nri3787 25614319 10.1038/nri3787 PMC7162685

[2] Crouse J, Kalinke U, Oxenius A (2015) Regulation of antiviral T cell responses by type I interferons. Nat Rev Immunol 15: 231–242. doi: 10.1038/nri3806 25790790 10.1038/nri3806

[3] Paquette RL, Hsu NC, Kiertscher SM, Park AN, Tran L, Roth MD et al (1998) Interferon-alpha and granulocyte-macrophage colony-stimulating factor differentiate peripheral blood monocytes into potent antigen-presenting cells. J Leukoc Biol 64: 358–367. 9738663 10.1002/jlb.64.3.358

[4] Parlato S, Santini SM, Lapenta C, Di Pucchio T, Logozzi M, Spada M et al (2001) Expression of CCR-7, MIP-3beta, and Th-1 chemokines in type I IFN-induced monocyte-derived dendritic cells: importance for the rapid acquisition of potent migratory and functional activities. Blood 98: 3022–3029. 11698286 10.1182/blood.v98.10.3022

[5] Spadaro F, Lapenta C, Donati S, Abalsamo L, Barnaba V, Belardelli F et al (2012) IFN-alpha enhances cross-presentation in human dendritic cells by modulating antigen survival, endocytic routing, and processing. Blood 119: 1407–1417. doi: 10.1182/blood-2011-06-363564 22184405 10.1182/blood-2011-06-363564

[6] Nguyen KB, Salazar-Mather TP, Dalod MY, Van Deusen JB, Wei XQ, Liew FY et al (2002) Coordinated and distinct roles for IFN-alpha beta, IL-12, and IL-15 regulation of NK cell responses to viral infection. J Immunol 169: 4279–4287. 12370359 10.4049/jimmunol.169.8.4279

[7] Brinkmann V, Geiger T, Alkan S, Heusser CH (1993) Interferon-Alpha Increases the Frequency of Interferon-Gamma-Producing Human Cd4+ T-Cells. J Exp Med 178: 1655–1663. 8228812 10.1084/jem.178.5.1655 PMC2191249

[8] Davidson S, Crotta S, McCabe TM, Wack A (2014) Pathogenic potential of interferon alphabeta in acute influenza infection. Nat Commun 5: 3864 doi: 10.1038/ncomms4864 24844667 10.1038/ncomms4864 PMC4033792

[9] Teijaro JR, Ng C, Lee AM, Sullivan BM, Sheehan KCF, Welch M et al (2013) Persistent LCMV Infection Is Controlled by Blockade of Type I Interferon Signaling. Science 340: 207–211. doi: 10.1126/science.1235214 23580529 10.1126/science.1235214 PMC3640797

[10] Wilson EB, Yamada DH, Elsaesser H, Herskovitz J, Deng J, Cheng G et al (2013) Blockade of chronic type I interferon signaling to control persistent LCMV infection. Science 340: 202–207. doi: 10.1126/science.1235208 23580528 10.1126/science.1235208 PMC3704950

[11] Zhen A, Rezek V, Youn C, Lam B, Chang N, Rick J et al (2017) Targeting type I interferon-mediated activation restores immune function in chronic HIV infection. J Clin Invest 127: 260–268. doi: 10.1172/JCI89488 27941243 10.1172/JCI89488 PMC5199686

[12] Murira A, Lamarre A (2016) Type-I Interferon Responses: From Friend to Foe in the Battle against Chronic Viral Infection. Front Immunol 7: 609 doi: 10.3389/fimmu.2016.00609 28066419 10.3389/fimmu.2016.00609 PMC5165262

[13] Enomoto H, Nishiguchi S (2015) Factors associated with the response to interferon-based antiviral therapies for chronic hepatitis C. World J Hepatol 7: 2681–2687. doi: 10.4254/wjh.v7.i26.2681 26609345 10.4254/wjh.v7.i26.2681 PMC4651912

[14] Azzoni L, Foulkes AS, Papasavvas E, Mexas AM, Lynn KM, Mounzer K et al (2013) Pegylated Interferon alfa-2a monotherapy results in suppression of HIV type 1 replication and decreased cell-associated HIV DNA integration. J Infect Dis 207: 213–222. doi: 10.1093/infdis/jis663 23105144 10.1093/infdis/jis663 PMC3532820

[15] Parker BS, Rautela J, Hertzog PJ (2016) Antitumour actions of interferons: implications for cancer therapy. Nat Rev Cancer 16: 131–144. doi: 10.1038/nrc.2016.14 26911188 10.1038/nrc.2016.14

[16] Hsu YC, Ho HJ, Wu MS, Lin JT, Wu CY (2013) Postoperative Peg-Interferon Plus Ribavirin Is Associated With Reduced Recurrence of Hepatitis C Virus-Related Hepatocellular Carcinoma. Hepatol 58: 150–157. 10.1002/hep.26300 23389758

[17] Reig M, Marino Z, Perello C, Inarrairaegui M, Ribeiro A, Lens S et al (2016) Unexpected high rate of early tumor recurrence in patients with HCV-related HCC undergoing interferon-free therapy. J Hepatol 65: 719–726. doi: 10.1016/j.jhep.2016.04.008 27084592 10.1016/j.jhep.2016.04.008

[18] Ivashkiv LB, Donlin LT (2014) Regulation of type I interferon responses. Nat Rev Immunol 14: 36–49. doi: 10.1038/nri3581 24362405 10.1038/nri3581 PMC4084561

[19] Imran M, Manzoor S, Ashraf J, Khalid M, Tariq M, Khaliq HM et al (2013) Role of viral and host factors in interferon based therapy of hepatitis C virus infection. Virol J 10: 299 doi: 10.1186/1743-422X-10-299 24079723 10.1186/1743-422X-10-299 PMC3849893

[20] Shin EC, Sung PS, Park SH (2016) Immune responses and immunopathology in acute and chronic viral hepatitis. Nat Rev Immunol 16: 509–523. doi: 10.1038/nri.2016.69 27374637 10.1038/nri.2016.69

[21] Chang Q, Wang YK, Zhao Q, Wang CZ, Hu YZ, Wu BY (2012) Th17 cells are increased with severity of liver inflammation in patients with chronic hepatitis C. J Gastroenterol Hepatol 27: 273–278. doi: 10.1111/j.1440-1746.2011.06782.x 21592230 10.1111/j.1440-1746.2011.06782.x

[22] Griffiths SJ, Dunnigan CM, Russell CD, Haas JG (2015) The Role of Interferon-lambda Locus Polymorphisms in Hepatitis C and Other Infectious Diseases. J Innate Immun 7: 231–242. doi: 10.1159/000369902 25634147 10.1159/000369902 PMC6738896

[23] Hwang Y, Kim W, Kwon SY, Yu HM, Kim JH, Choe WH (2015) Incidence of and risk factors for thyroid dysfunction during peginterferon alpha and ribavirin treatment in patients with chronic hepatitis C. Korean J Intern Med 30: 792–800. doi: 10.3904/kjim.2015.30.6.792 26552454 10.3904/kjim.2015.30.6.792 PMC4642008

[24] Joo SK, Kim JH, Oh S, Kim BG, Lee KL, Kim HY et al (2015) Prospective Comparison of Noninvasive Fibrosis Assessment to Predict Advanced Fibrosis or Cirrhosis in Asian Patients With Hepatitis C. J Clin Gastroenterol 49: 697–704. doi: 10.1097/MCG.0000000000000215 25203365 10.1097/MCG.0000000000000215

[25] Griffiths SJ, Dunnigan CM, Russell CD, Haas JG (2015) The Role of Interferon-lambda Locus Polymorphisms in Hepatitis C and Other Infectious Diseases. J Innate Immun 7: 231–242. doi: 10.1159/000369902 25634147 10.1159/000369902 PMC6738896

[26] Jimenez-Sousa MA, Almansa R, de la Fuente C, Caro-Paton A, Ruiz L, Sanchez-Antolín G et al (2010) Increased Th1, Th17 and pro-fibrotic responses in hepatitis C-infected patients are down-regulated after 12 weeks of treatment with pegylated interferon plus ribavirin. Eur Cytokine Netw 21: 84–91. doi: 10.1684/ecn.2010.0191 20483710 10.1684/ecn.2010.0191

[27] Meng P, Zhao S, Niu X, Fu N, Su S, Wang R et al. (2016) Involvement of the Interleukin-23/Interleukin-17 Axis in Chronic Hepatitis C Virus Infection and Its Treatment Responses. Int J Mol Sci 17. 10.3390/ijms17071070 PMC4964446 27428948

[28] Hao C, Zhou Y, He Y, Fan C, Sun L, Wei X et al (2014) Imbalance of regulatory T cells and T helper type 17 cells in patients with chronic hepatitis C. Immunol 143: 531–538. 10.1111/imm.12330 PMC4253501 24903732

[29] Kim BS, Park YJ, Chung Y (2016) Targeting IL-17 in autoimmunity and inflammation. Arch Pharm Res 39: 1537–1547. doi: 10.1007/s12272-016-0823-8 27576555 10.1007/s12272-016-0823-8

[30] Beringer A, Noack M, Miossec P (2016) IL-17 in Chronic Inflammation: From Discovery to Targeting. Trends Mol Med 22: 230–241. doi: 10.1016/j.molmed.2016.01.001 26837266 10.1016/j.molmed.2016.01.001

[31] Ferenci P, Fried MW, Shiffman ML, Smith CI, Marinos G, Gonçales FL Jr et al (2005) Predicting sustained virological responses in chronic hepatitis C patients treated with peginterferon alfa-2a (40 KD)/ribavirin. J Hepatol 43: 425–433. doi: 10.1016/j.jhep.2005.04.009 15990196 10.1016/j.jhep.2005.04.009

[32] Bressler BL, Guindi M, Tomlinson G, Heathcote J (2003) High body mass index is an independent risk factor for nonresponse to antiviral treatment in chronic hepatitis C. Hepatol 38: 639–644. 10.1053/jhep.2003.50350 12939590

[33] Dogan UB, Akin MS, Yalaki S (2013) Alanine aminotransferase normalization at week 8 predicts viral response during hepatitis C treatment. World J Gastroenterol 19: 8678–8686. doi: 10.3748/wjg.v19.i46.8678 24379586 10.3748/wjg.v19.i46.8678 PMC3870514

[34] Jung YK, Kim JH, Ahn SM, Yang JW, Park SJ, Kim JW et al (2013) Role of interleukin 28B-related gene polymorphisms in chronic hepatitis C and the response to antiviral therapy in Koreans. J Clin Gastroenterol 47: 644–650. doi: 10.1097/MCG.0b013e3182896abf 23442843 10.1097/MCG.0b013e3182896abf

[35] Heo NY, Lim YS, Lee W, Oh M, An J, Lee D et al (2014) No association between the IL28B SNP and response to peginterferon plus ribavirin combination treatment in Korean chronic hepatitis C patients. Clin Mol Hepatol 20: 177–184. doi: 10.3350/cmh.2014.20.2.177 25032184 10.3350/cmh.2014.20.2.177 PMC4099333

[36] Prokunina-Olsson L, Muchmore B, Tang W, Pfeiffer RM, Park H, Dickensheets H et al (2013) A variant upstream of IFNL3 (IL28B) creating a new interferon gene IFNL4 is associated with impaired clearance of hepatitis C virus. Nat Genet 45: 164–171. doi: 10.1038/ng.2521 23291588 10.1038/ng.2521 PMC3793390

[37] Jung YK, Kim JH (2013) Is peginterferon and ribavirin therapy effective in Korean patients with chronic hepatitis C? Clin Mol Hepatol 19: 26–28. doi: 10.3350/cmh.2013.19.1.26 23593606 10.3350/cmh.2013.19.1.26 PMC3622852

[38] Lee HC, Sung SSJ, Krueger PD, Jo YA, Rosen HR, Ziegler SF et al (2013) Hepatitis C virus promotes t-helper (Th)17 responses through thymic stromal lymphopoietin production by infected hepatocytes. Hepatol 57: 1314–1324. 10.1002/hep.26128 PMC3582737 23150092

[39] Loftis JM, Morasco BJ, Menasco D, Fuchs D, Strater M, Hauser P (2010) Serum Serotonin Levels are Associated with Antiviral Therapy Outcomes in Patients with Chronic Hepatitis C. Open Infect Dis J 4: 132–141. 21151716 10.2174/1874279301004010132 PMC2999909

[40] Lagging M, Romero AI, Westin J, Norkrans G, Dhillon AP, Pawlotsky JM et al (2006) IP-10 predicts viral response and therapeutic outcome in difficult-to-treat patients with HCV genotype 1 infection. Hepatol 44: 1617–1625. 10.1002/hep.21407 17133471

[41] Shin HM, Lee JW, Cho NH (2015) Role of Th17 and Treg during the Chronic Infection of Hepatitis C Virus. J Bacteriol Virol 45: 389–393.

[42] Reda R, Abbas AA, Mohammed M, El Fedawy SF, Ghareeb H, El Kabarity RH et al (2015) The Interplay between Zinc, Vitamin D and, IL-17 in Patients with Chronic Hepatitis C Liver Disease. J Immunol Res 846348. 10.1155/2015/846348 PMC4609465 26504859

[43] Sousa GM, Oliveira IS, Andrade LJ, Sousa-Atta ML, Parana R, Atta AM (2012) Serum levels of Th17 associated cytokines in chronic hepatitis C virus infection. Cytokine 60: 138–142. doi: 10.1016/j.cyto.2012.06.003 22748467 10.1016/j.cyto.2012.06.003

[44] Romero AI, Lagging M, Westin J, Dhillon AP, Dustin LB, Pawlotsky JM et al (2006) Interferon (IFN)-gamma-inducible protein-10: association with histological results, viral kinetics, and outcome during treatment with pegylated IFN-alpha 2a and ribavirin for chronic hepatitis C virus infection. J Infect Dis 194: 895–903. doi: 10.1086/507307 16960776 10.1086/507307

[45] Kim CH, Kim JH, Jong YK, Kim JH, Choi JH, Hong SK et al (2008) The clinical significance of interferon inducible protein-10 in chronic hepatitis C patients with genotype I undergoing pegylated interferon and ribavirin therapy. J Gastroenterol and Hepatol 23: A42–A42.

[46] Sandhu DS, Baichoo E, Roberts LR (2014) Fibroblast growth factor signaling in liver carcinogenesis. Hepatol 59: 1166–1173. 10.1002/hep.26679 24716202

[47] Kang W, Shin EC (2011) Clinical implications of chemokines in acute and chronic hepatitis C virus infection. Yonsei Med J 52: 871–878. doi: 10.3349/ymj.2011.52.6.871 22028149 10.3349/ymj.2011.52.6.871 PMC3220267

[48] Noack M, Miossec P (2014) Th17 and regulatory T cell balance in autoimmune and inflammatory diseases. Autoimmun Rev 13: 668–677. doi: 10.1016/j.autrev.2013.12.004 24418308 10.1016/j.autrev.2013.12.004

[49] Rajoriya N, Fergusson JR, Leithead JA, Klenerman P (2014) Gamma Delta T-lymphocytes in Hepatitis C and Chronic Liver Disease. Front Immunol 5: 400 doi: 10.3389/fimmu.2014.00400 25206355 10.3389/fimmu.2014.00400 PMC4143964

[50] Jie ZL, Liang YJ, Hou LF, Dong C, Iwakura Y, Soong L et al (2014) Intrahepatic Innate Lymphoid Cells Secrete IL-17A and IL-17F That Are Crucial for T Cell Priming in Viral Infection. J Immunol 192: 3289–3300. doi: 10.4049/jimmunol.1303281 24600029 10.4049/jimmunol.1303281 PMC3967589

