# Supplementary material for: Immunological dynamics associated with rapid virological response during the early phase of type I interferon therapy in patients with chronic hepatitis C
Source: PLoS One. 2017 Jun 14;12(6):e0179094. doi: 10.1371/journal.pone.0179094 (PMC5470700; doi:10.1371/journal.pone.0179094)

Supporting information for

Immunological dynamics associated with rapid virological response during the early phase of type I interferon therapy in patients with chronic hepatitis C

Jae-Won Lee[1,2], Won Kim[3,*], Eun-Kyung Kwon[1,2], Yuri Kim[1,2], Hyun Mu Shin[2], Dong-Hyun Kim[1,2], Chan-Ki Min[1,2], Ji-Yeob Choi[2,4], Won-Woo Lee[1,2,4], Myung-Sik Choi[1,5], Byeong Gwan Kim[3], and Nam-Hyuk Cho[1,2,5,*]

[1]Department of Microbiology and Immunology, Seoul National University College of Medicine, Seoul, Republic of Korea

[2]Department of Biomedical Science, Seoul National University College of Medicine, Seoul, Republic of Korea

[3]Department of Internal Medicine, Seoul National University College of Medicine, Seoul Metropolitan Government Boramae Medical Center, Seoul, Republic of Korea

[4]Cancer Research Institute, Seoul National University College of Medicine, Seoul, Republic of Korea.

[5]Institute of Endemic Disease, Seoul National University Medical Research Center and Bundang Hospital, Seoul, Republic of Korea

* Corresponding authors

E-mail: drwon1@snu.ac.kr (WK), chonh@snu.ac.kr (NHC)

S Figure 1. Gating strategies for the analysis of diverse immune cell types in peripheral blood mononuclear cells of chronic hepatitis C patients. (A) NK cells and CD4 T Cells expressing IL-17A. (B) CD8$^-$/CD4$^+$/CD25$^+$/CD19$^-$/FoxP3$^+$ regulatory T cells. (C) Lin$^-$/IL-7R$^+$/CD45$^+$/IL13$^+$ group 2 innate lymphoid cells and Lin$^-$/IL-7R$^+$/CD45$^+$/IL17$^+$ group 3 innate lymphoid cells.

A)

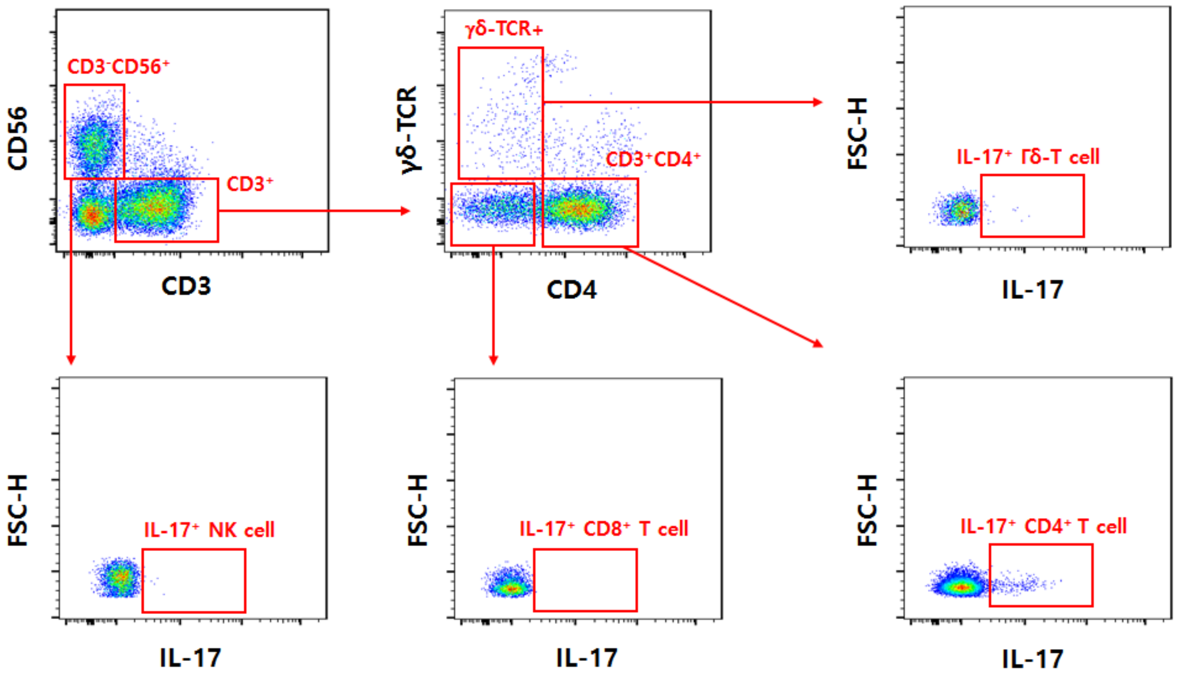

B)

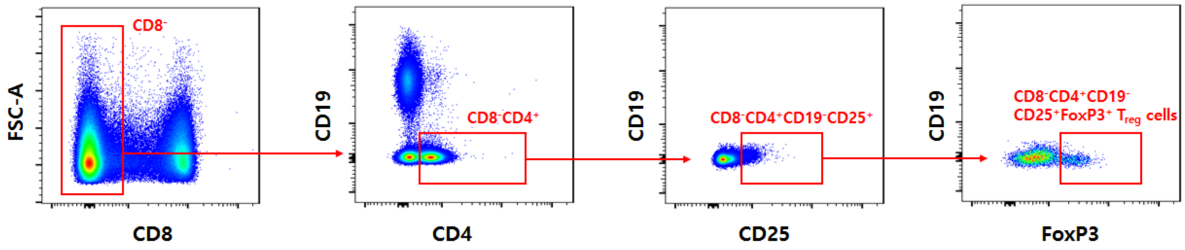

C)

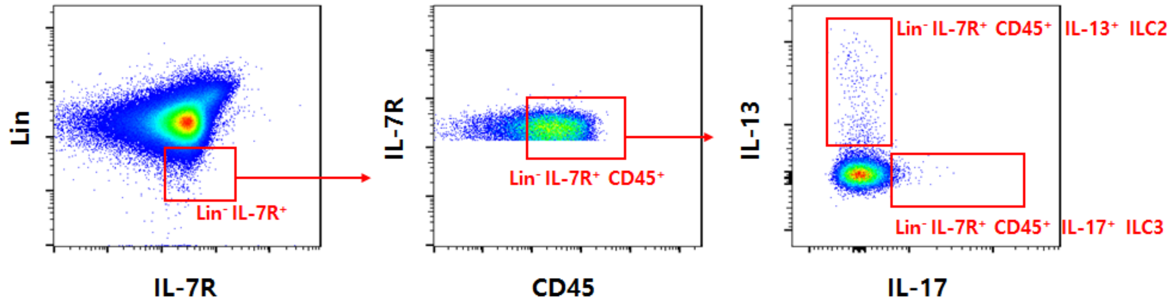

Supplement: S1 Fig — (PDF) [file pone.0179094.s001.pdf]
